# Clinical application of abdominal breathing training and evaluation of physical and mental benefits in anxiety patients

**DOI:** 10.3389/fpsyg.2025.1695622

**Published:** 2025-12-09

**Authors:** Jiajin Chai, Shikun Li, Li He, Jiayuan Yang, Ting Wu, Kunyang Ou, Xichen Chen, Kaicheng Ma, Shouping Zhao

**Affiliations:** 1 Integrative Medicine Department, The Affiliated Mental Health Center of Kunming Medical University, Kunming, Yunnan, China; 2 Department of Psychiatry, The Affiliated Mental Health Center of Kunming Medical University, Kunming, Yunnan, China; 3 The First Affiliated Hospital of Kunming Medical University, Kunming, Yunnan, China; 4 Outpatient and Emergency Department, The Affiliated Mental Health Center of Kunming Medical University, Kunming, Yunnan, China; 5 Department of Geriatric Psychiatry, The Affiliated Mental Health Center of Kunming Medical University, Kunming, Yunnan, China

**Keywords:** anxiety, breathing training, RCT, physiological, abdominal breathing

## Abstract

**Background:**

Anxiety disorders represent highly prevalent mental health conditions globally (lifetime prevalence: ~7–14%). Current pharmacological treatments carry risks of dependence and metabolic side effects, while psychological therapies face accessibility limitations. There is an urgent need to develop safe, accessible non-pharmacological interventions. This single-blind randomized controlled trial systematically evaluated the efficacy and sustainability of an 8-week standardized abdominal breathing training program in alleviating anxiety symptoms and modulating autonomic nervous function in patients with anxiety disorders.

**Methods:**

A total of 120 outpatient participants (aged 18–65 years) meeting ICD-10 diagnostic criteria for Generalized Anxiety Disorder were recruited. Participants were randomly assigned to either an intervention group (abdominal breathing training, *n* = 60) or a control group (treatment-as-usual, *n* = 60). The intervention group received professionally supervised training consisting of 3–4 daily sessions of 10–15 min each (inhale:exhale ratio = 1:2; inhalation 3–5 s/exhalation 5–7 s). Adherence was monitored via respiratory sensors (mean adherence rate: 92.4%). Assessments using the Self-Rating Anxiety Scale (SAS) and physiological measurements—including heart rate (HR), respiratory rate (RR), blood pressure (BP), and heart rate variability (HRV-LF/HF ratio)—were conducted at baseline, post-intervention (8 weeks), and at 4-week follow-up (intervention group only).

**Results:**

*Anxiety symptom improvement*: Two-way repeated measures ANOVA revealed a significant group × time interaction effect on SAS scores (*p* < 0.05). Post-intervention, the intervention group exhibited a significant reduction in SAS scores compared to baseline (67.57 ± 8.88 vs. 71.80 ± 3.99, *p* < 0.001), reflecting a 5.9% decrease, and scores were significantly lower than the control group (70.43 ± 4.33, *p* = 0.027). At 4-week follow-up, despite a slight rebound, SAS scores in the intervention group remained significantly below baseline (68.57 ± 8.11, *p* < 0.05). *Physiological optimization*: For physiological indicators, significant group × time interaction effects were observed (all *p* < 0.05). Post-intervention, the intervention group showed significant reductions in heart rate (77.08 ± 10.30 vs. 83.37 ± 9.67 bpm), respiratory rate (17.37 ± 1.78 vs. 18.60 ± 1.83 breaths/min), systolic blood pressure (114.12 ± 11.97 vs. 122.63 ± 12.18 mmHg), and diastolic blood pressure (74.40 ± 6.75 vs. 80.28 ± 7.58 mmHg) (all *p* < 0.05). Concurrently, the HRV-LF/HF ratio increased significantly (*p* = 0.008).

**Conclusion:**

Standardized abdominal breathing training significantly alleviates anxiety symptoms (effect size Cohen’s d = 0.61) and induces sustained physiological improvements in autonomic regulation (effects maintained 4 weeks post-intervention). As a cost-free, non-pharmacological intervention devoid of adverse effects, it provides an effective adjunctive treatment option for anxiety disorders, particularly benefiting medically underserved populations and individuals with medication intolerance, suggesting significant clinical potential.

## 1 Introduction

Anxiety disorder, also known as anxiety, is a common mental illness characterized by excessive worry, fear, and tension (Zeng et al., 2025; Lai et al., 2023). The main symptoms of anxiety disorder include persistent worry and fear, feelings of anxiety and tension, accelerated heart rate or chest tightness, difficulty breathing, insomnia, and concentration difficulties (DeGeorge et al., 2022). Statistics show that globally, the prevalence rate of anxiety disorder fluctuates between 7.3 and 28.0% (GBD 2019 Mental Disorders Collaborators, 2022), while in China, the lifetime prevalence rate reaches 7.6% (Yu et al., 2018). It is worth noting that the World Health Organization has explicitly listed anxiety disorder as one of the main causes of disability worldwide, ranking sixth, which undoubtedly highlights its significant impact on public health (Baxter et al., 2014). A recent study published in The Lancet by researchers from the University of Washington in the United States and the University of Queensland in Australia shows that in 2020, the global rates of major depressive disorder and anxiety disorder increased by 28 and 26%, respectively (COVID-19 Mental Disorders Collaborators, 2021). At the same time, anxiety disorder often co-occurs with other mental illnesses (Kessler et al., 2005). Therefore, finding simple and effective methods to improve depressive and anxious emotional disorders is particularly important.

Currently, the treatment of anxiety disorder primarily involves medication, psychological therapy, and physical therapy (Walder et al., 2025). Commonly used medications include SNRIs, SSRIs, 5-hydroxytryptamine 1A receptor partial agonists, and benzodiazepines, which are recommended as first-line drugs for treating anxiety disorder (Vu and Conant-Norville, 2021; Mishra and Varma, 2023). Studies show that benzodiazepines can only temporarily relieve anxiety symptoms, and patients taking these medications experience varying degrees of side effects (Fang et al., 2025). Research indicates that up to 38% of individuals who take anti-anxiety medications long-term experience varying degrees of cognitive/physical side effects. In 2020, the U.S. FDA warned that taking benzodiazepines for more than 2 weeks can easily lead to addiction, physical dependence, and withdrawal symptoms (Pergolizzi et al., 2021). In psychological therapy, CBT is recognized as a first-line treatment method, helping patients improve their anxiety and depressive emotions (David et al., 2018; Ren et al., 2025). Although psychological therapy is effective, resources are relatively limited and costly. Related studies suggest that the advantages of CBT may not be as significant as expected for certain subtypes or patients with milder symptoms (Simon et al., 2021). From the perspective of long-term efficacy, side effect burden, and accessibility of medical resources, it is urgent to develop low-cost, low-risk non-pharmacological intervention strategies that can serve as supplements or alternatives.

In this context, respiratory regulation training, particularly abdominal breathing, has increasingly attracted the attention of researchers (Liu et al., 2011). Abdominal breathing is a breathing method primarily characterized by the movement of the diaphragm (Salah et al., 2022). During inhalation, the diaphragm descends, causing the abdomen to protrude outward, allowing more air to enter the lungs; during exhalation, the diaphragm rises, and the abdomen contracts, expelling air from the lungs (Zhang et al., 2012). Abdominal breathing can regulate autonomic nervous system function (Wang et al., 2006). When performing abdominal breathing, it stimulates the parasympathetic nervous system, enhancing its excitability and inhibiting excessive activation of the sympathetic nervous system, thereby modulating the body’s stress response and alleviating physiological symptoms such as accelerated heart rate and elevated blood pressure caused by anxiety (Billman, 2013; Gerritsen and Band, 2018; Zaccaro et al., 2018). Although preliminary studies indicate that breathing training, especially abdominal breathing, is beneficial for emotional regulation (Jerath et al., 2015), more high-quality evidence is needed to support its systematic and standardized application effects and quantified assessment of mind–body benefits in clinical anxiety patient populations.

However, existing research has critical limitations. First, there is heterogeneity in efficacy: some studies observe increased HRV (Ma et al., 2017; Shaffer and Ginsberg, 2017), yet others fail to detect changes in cortisol (a key stress hormone) (Hu et al., 2024). Second, intervention protocols lack standardization—variations in training duration (10 days to 8 weeks) and respiratory rate (6–12 breaths per minute) hinder cross-study comparisons (Chen et al., 2017; Czub et al., 2024). Third, adherence monitoring is inadequate: most studies do not use objective tools like sensors, and long-term follow-up data are scarce, making it impossible to confirm the sustainability of effects (Hu et al., 2024)—a key issue the present study aims to address through its design.

Abdominal breathing modulates the autonomic nervous system (enhancing parasympathetic activity and inhibiting sympathetic overactivation) (Zaccaro et al., 2018), thereby alleviating anxiety-related physiological symptoms such as accelerated heart rate and elevated blood pressure. Despite preliminary evidence supporting its value (Jerath et al., 2015), the aforementioned limitations highlight the need for standardized randomized controlled trials (RCTs)—such as the design of this study—to further validate the efficacy and sustainability of diaphragmatic breathing in patients with anxiety.

Based on the above background, this study aims to systematically evaluate the improvement effects of an 8-week standardized abdominal breathing training program on anxiety symptoms and key physiological indicators in anxiety patients through a randomized controlled trial (RCT), and to track its short-term persistence, providing empirical evidence for the clinical application of abdominal breathing in anxiety management.

## 2 Methods

### 2.1 Study design

Adopted a randomized controlled trial design. Using the random number table method, 120 research subjects who met the inclusion criteria were divided into an experimental group and a control group, with 60 subjects in each group. The experimental group received 8 weeks of abdominal breathing training, while the control group received no intervention. Anxiety level assessments and physiological parameter measurements were conducted on both groups before and after the experiment, and a follow-up evaluation was performed on the experimental group 4 weeks after the intervention ended.

### 2.2 Research subjects

Individuals who sought help at the Integrated Traditional Chinese and Western Medicine Department of Yunnan Provincial Psychiatric Hospital and were assessed as having varying degrees of anxiety based on the Self-Rating Anxiety Scale (SAS).

#### 2.2.1 Inclusion criteria

meeting the diagnostic criteria for Anxiety Disorder in the International Classification of Diseases, 10th Revision - Mental and Behavioral Disorders (ICD-10) and being newly diagnosed cases; aged 18 to 60 years; no history of severe cardiovascular, respiratory, or neurological diseases; having not received pharmacological or psychological treatment in the past 3 months; voluntarily participating in this study and signing the informed consent form.

#### 2.2.2 Exclusion criteria

Received anti-anxiety medication or psychological treatment within the recent 3 months; cognitive impairment that prevents cooperation with researchers. A total of 120 study subjects were included, with 60 in the experimental group and 60 in the control group. There were no statistically significant differences between the two groups in terms of age, gender, educational level, and baseline SAS scores (*p* > 0.05), making them comparable.

### 2.3 Intervention measures

#### 2.3.1 Experimental group

Guided by professionally trained personnel to perform abdominal breathing training. First, the experimental group members were given a detailed explanation of the principles, methods, and precautions of abdominal breathing. During training, they could choose to lie flat on their bed, sit in a comfortable chair, or stand. Breathe in slowly through the nose, focusing attention on the abdomen, feeling the abdomen expand like a balloon, keeping the chest as still as possible, with the inhalation duration lasting 3–5 s; then exhale slowly through the mouth, feeling the abdomen gradually contract like a balloon deflating, with the exhalation duration lasting 5–7 s. Initially, practice 2–3 times a day, each time lasting 5–15 min; as proficiency increases, gradually increase the number of sets and duration of practice, ultimately reaching 3–4 sets a day, each set lasting 10–15 min. During the training process, the experimental group members are required to maintain a relaxed mindset, and can adjust the breathing rhythm or pause the training if they feel uncomfortable.

#### 2.3.2 Control group

No intervention measures related to abdominal breathing are performed, but the control group members are informed to maintain normal life during the study period, avoiding deliberate engagement in other activities or treatments that may affect anxiety status.

### 2.4 Measurement tools and indicators

#### 2.4.1 Self-Rating Anxiety Scale (SAS)

The Self-Rating Anxiety Scale developed by Zung (1971) and Dunstan et al. (2017) is adopted. Its most prominent feature is its simplicity, time-saving nature, and ease of mastery, enabling it to quickly reflect the subjective anxiety level perceived by the respondent and thus rapidly evaluate an individual’s anxiety status. Regarded as a convenient, efficient, cost-effective, and highly reliable psychological assessment tool, the SAS is widely applied in fields such as medical care, education, and scientific research. This scale consists of 20 items, each scored on a 1–4 scale, primarily assessing the frequency of symptoms defined by the items. The raw score is obtained by summing the scores of the 20 items. The standard score is obtained by multiplying the raw score by 1.25 and taking the integer part. A standard score below 50 is considered normal; 50–59 indicates mild anxiety; 60–69 indicates moderate anxiety; and 70 or above indicates severe anxiety. The SAS assessment is conducted for both groups before the experiment, after the experiment, and during follow-up for the experimental group.

#### 2.4.2 Physiological indicator measurement

A multi-parameter physiological monitoring device is used to measure heart rate, respiratory rate, and blood pressure of the two groups of research subjects in a quiet state. Before measurement, the research subjects need to rest quietly for more than 15 min to ensure the accuracy of the measurement results. The physiological indicator measurement is conducted for both groups before and after the experiment.

### 2.5 Data collection and analysis

Data were collected by uniformly trained data collectors in accordance with standardized procedures to ensure the accuracy and completeness of the data. A double-entry verification method was adopted for data entry to prevent data entry errors. Statistical analysis was performed using SPSS 26.0 software. Quantitative data were expressed as mean ± standard deviation (x ± s). Comparisons between the two groups were conducted using an independent samples *t*-test, while intragroup pre- and post-intervention comparisons were made using a paired samples *t*-test. Two-way repeated measures analysis of variance (ANOVA) was employed to examine the main effects of time and group as well as their interaction effect. For the SAS scores of the experimental group at three time points (pre-intervention, post-intervention, and follow-up), one-way repeated measures ANOVA was applied. Categorical data were presented as counts and percentages, and intergroup comparisons were performed using the Chi-square test. A *p*-value < 0.05 was considered statistically significant.

## 3 Research results

**Figure 1:**
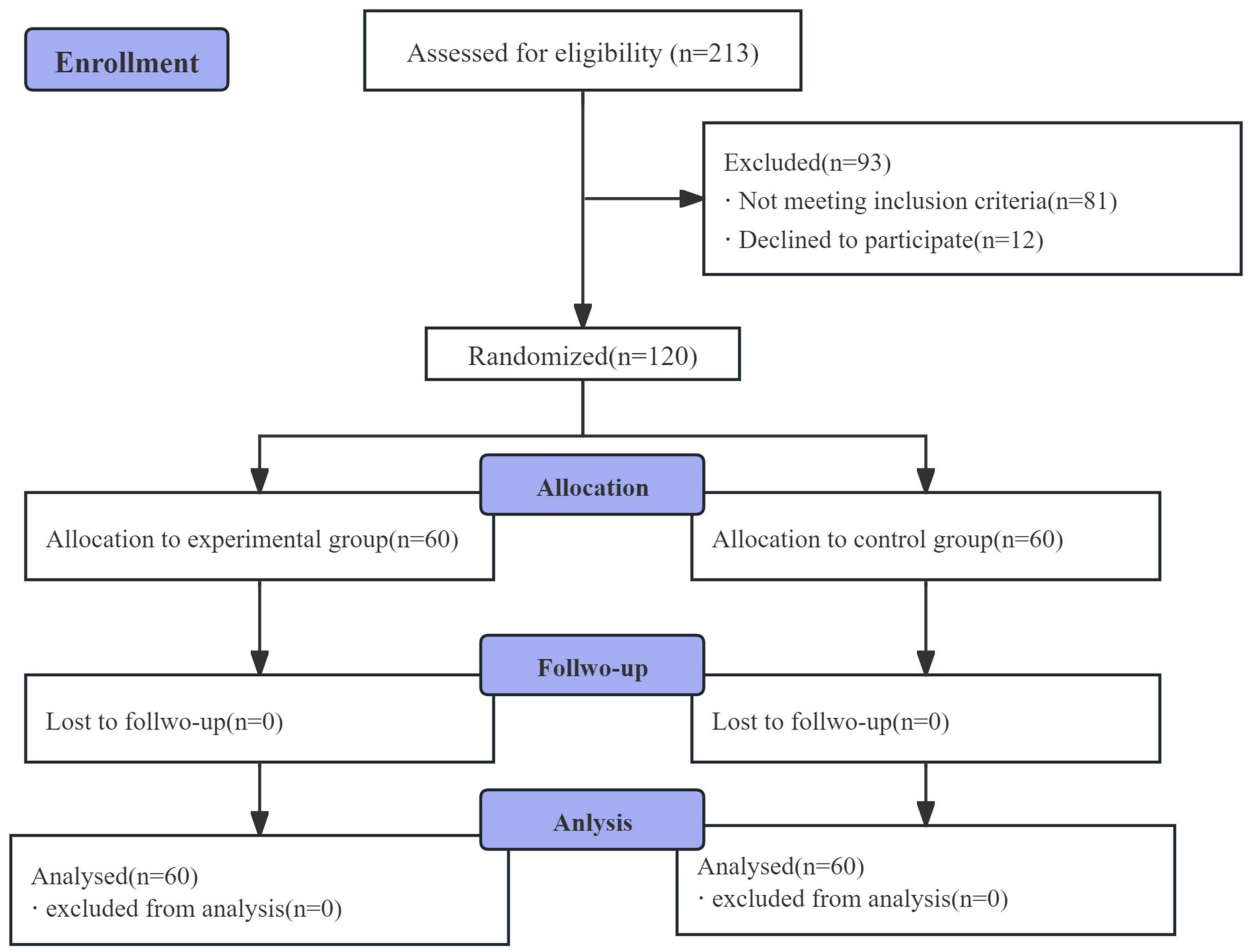
Flowchart of enrollment, allocation, and data analysis.

A total of 213 participants underwent eligibility screening, of whom 120 were enrolled into the trial. Eighty-one did not satisfy the inclusion criteria, and 12 declined participation. Ultimately, all 120 participants received the assigned intervention, were included in the statistical analysis, and completed the study, as shown in Figure 1.

### 3.1 Comparison of general data before experiment between the two groups

There were no statistically significant differences between the experimental group and the control group in terms of general data such as age, gender, educational level, as well as pre-experiment SAS scores, heart rate, respiratory rate, and blood pressure (*p* > 0.05). The specific data are shown in Table 1.

**Table 1:** Comparison of general information and baseline indicators before the two groups of experiments.

Title	name	Experimental group (*n* = 60)	Control group (*n* = 60)	*χ*^2^/*t*	*p*
Gender	Women	32 (53.333)	30 (50.000)	0.133	0.715
	Man	28 (46.667)	30 (50.000)		
Education Level	Associate’s Degree	16 (26.667)	16 (26.667)	1.295	0.730
	Bachelor’s Degree	17 (28.333)	12 (20.000)		
	Master’s Degree and Above	14 (23.333)	17 (28.333)		
	High school (secondary vocational school) and below	13 (21.667)	15 (25.000)		
Age		39.317 ± 12.920	38.167 ± 12.061	0.504	0.615
Pre-experiment SAS score		71.809 ± 3.991	71.024 ± 3.953	1.083	0.281
Pre-experiment heart rate		83.367 ± 9.669	83.400 ± 9.937	−0.019	0.985
Respiratory rate before experiment		18.600 ± 1.834	18.250 ± 1.874	1.034	0.303
Systolic blood pressure before experiment		122.633 ± 12.178	123.167 ± 11.927	−0.242	0.809
Pre-experiment diastolic blood pressure		80.283 ± 7.596	80.933 ± 7.417	−0.474	0.636

Table 1 presents the general data and baseline indicator comparisons between the experimental group and the control group before the experiment. Statistical analysis indicates that there was no significant difference in gender composition between the two groups (*p* > 0.05); at the education level, the distribution of people across various levels including college diploma, undergraduate, master and above, high school (secondary vocational school) and below showed no statistically significant intergroup difference (*p* > 0.05). Additionally, in key physiological and psychological baseline indicators such as age, Self-Rating Anxiety Scale (SAS) score, heart rate, respiratory rate, systolic blood pressure, and diastolic blood pressure, the data of the two groups showed no significant differences (*p* > 0.05). This result fully demonstrates that before the experiment, the two groups of research subjects exhibited good balance and comparability in demographic characteristics and basic physiological and psychological indicators. This conclusion effectively excludes the potential interference of initial intergroup differences on subsequent experimental results, ensuring the scientific rigor of the research design, and lays a reliable data foundation for accurately evaluating the intervention effects of abdominal breathing training, making the research conclusions more persuasive and credible.

### 3.2 Comparison of SAS scores before and after the two groups of experiments

Two-way repeated measures ANOVA showed that the interaction effect between group and time was statistically significant (*p* < 0.05), indicating that the changes in patients’ SAS scores over time were affected by the intervention measures of different groups. Therefore, it is necessary to analyze the simple effects of group and time. The specific data are shown in Table 2.

**Table 2:** Comparison of SAS scores before and after the two groups of experiments and at the follow-up of the experimental group ( $\bar{x}$ ± s, scores).

SAS score	Experimental group (*n* = 60)	Control group (*n* = 60)	*t*	*p*
Before the experiment	71.80 ± 3.99	71.02 ± 3.95	1.081	0.282
Post-experiment	67.57 ± 8.88	70.43 ± 4.33	−2.246	0.027
t	3.435	0.735		
p	0.001	0.465		
Follow-up	68.57 ± 8.11			
F	9.562			
p	<0.001			

All comparisons are with pre-experiment values.

Before the experiment, there was no statistically significant difference in SAS scores between the two groups (*p* > 0.05); after the experiment, the SAS score of the experimental group was lower than that of the control group, and the difference was statistically significant (*p* < 0.05).

In the experimental group, the SAS score after the experiment was lower than that before the experiment, and the difference was statistically significant (*p* < 0.05); in the control group, there was no statistically significant difference in SAS scores before and after the experiment (*p* > 0.05).

**Figure 2:**
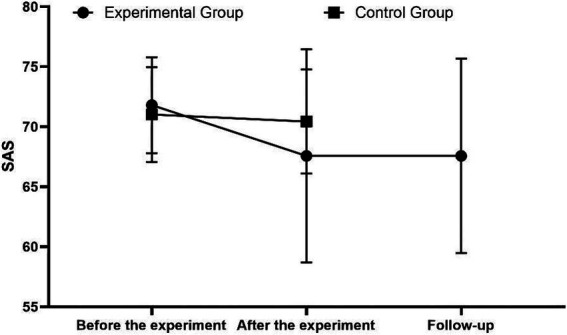
Comparison of SAS scores before and after the two groups of experiments and at the follow-up of the experimental group.

Further analysis of SAS scores of the experimental group at three time points was performed using one-way repeated measures ANOVA, which showed that there were statistically significant differences in SAS scores of the experimental group at the three time points (*p* < 0.05). Specific Bonferroni multiple comparisons revealed that the SAS scores of patients after the experiment and during follow-up were both lower than those before the experiment, and the differences were statistically significant (*p* < 0.05) (see Figure 2).

### 3.3 Comparison of physiological indicators before and after the experiment in the two groups

Two-way repeated measures analysis of variance (ANOVA) revealed that the interaction effect between group and time was statistically significant for heart rate (HR), respiratory rate (RR), systolic blood pressure (SBP), and diastolic blood pressure (DBP) (all *p* < 0.05). This indicates that the temporal changes in each of these physiological indicators among patients are influenced by the intervention measures implemented in different groups; thus, it is necessary to conduct simple effect analysis of group and time. The specific data are shown in Table 3.

**Table 3:** Comparison of physiological indicators before and after the experiment for the two groups ( $\bar{x}$ ± s).

Name	Experimental group		Control group	
	Before the experiment	Post-experiment	Before the experiment	Post-experiment
Heart rate	83.37 ± 9.67	77.08 ± 10.30^*#^	83.40 ± 9.93	83.28 ± 10.31
Respiratory rate in the	18.60 ± 1.83	17.37 ± 1.78^*#^	18.25 ± 1.87	18.05 ± 1.75
Systolic blood pressure	122.63 ± 12.18	114.12 ± 11.97^*#^	123.17 ± 11.90	122.48 ± 10.84
Diastolic blood pressure	80.28 ± 7.58	74.40 ± 6.75^*#^	80.93 ± 7.39	80.43 ± 7.60

Note: * *P* < 0.05 compared with the control group after the experiment; ^#^ *P* < 0.05 compared with the same group before the experiment.

Prior to the experiment, no statistically significant differences were observed in HR, RR, SBP, or DBP between the two groups (*p* > 0.05). Following the experiment, HR, RR, SBP, and DBP in the experimental group were significantly lower than those in the control group, with statistically significant differences (*p* < 0.05).

**Figure 3:**
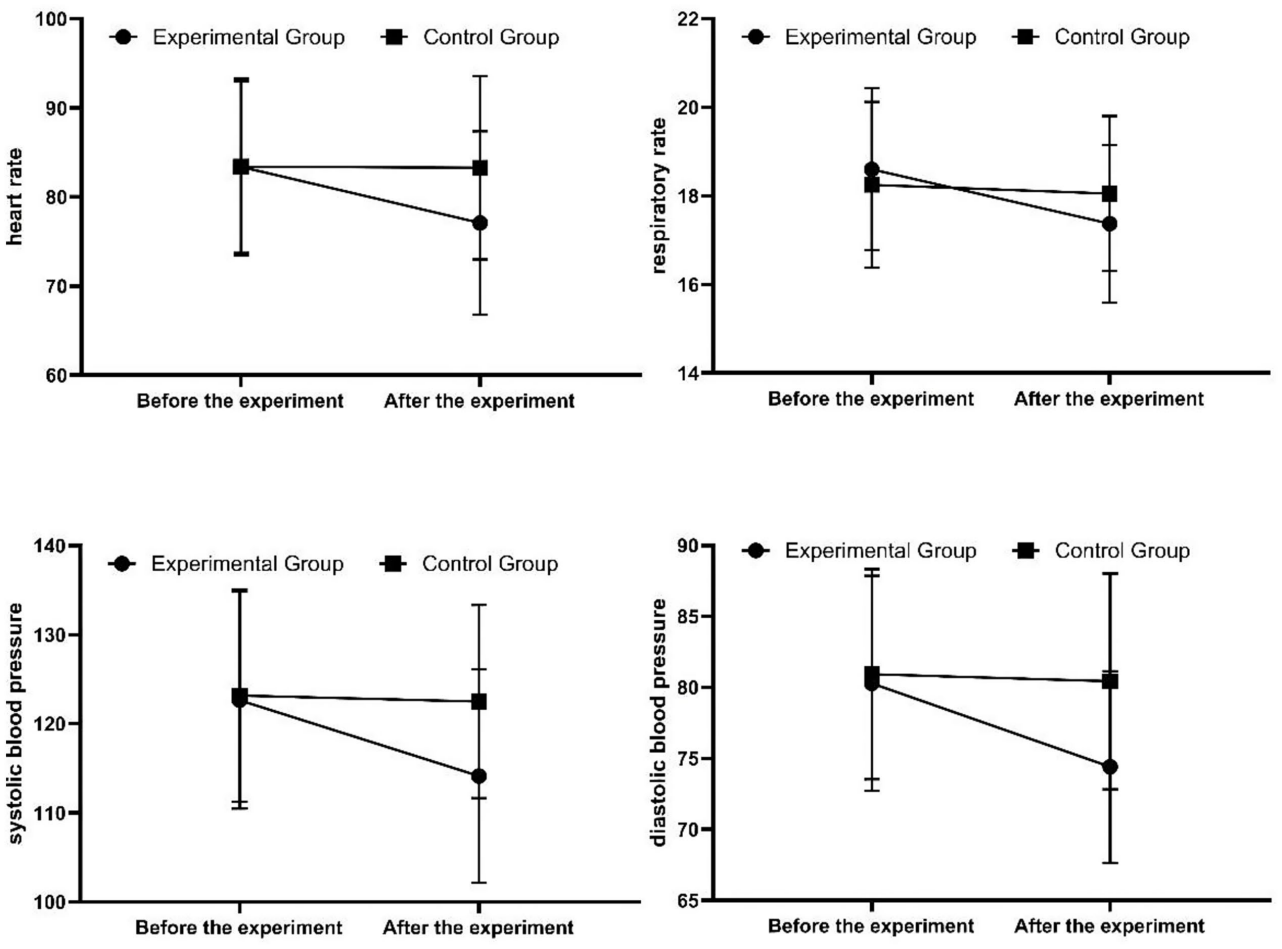
Comparison of physiological indicators before and after the experiment for the two groups.

In the experimental group, HR, RR, SBP, and DBP after the experiment were significantly lower than the pre-experiment values, and the differences were statistically significant (*p* < 0.05). In the control group, there were no statistically significant differences in HR, RR, SBP, or DBP between the pre-experiment and post-experiment periods (*p* > 0.05) (see Figure 3).

## 4 Discussion

### 4.1 Analysis of the effect of abdominal breathing on improving anxiety

The results of this study show that after 8 weeks of abdominal breathing training, the SAS scores of the experimental group significantly decreased, indicating that abdominal breathing can effectively reduce anxiety levels. This is consistent with previous related research findings (Ma et al., 2017; Hopper et al., 2019). Abdominal breathing regulates the autonomic nervous system, enhancing the excitability of the parasympathetic nervous system and inhibiting the excessive excitation of the sympathetic nervous system, thereby alleviating the physiological stress responses caused by anxiety, such as increased heart rate and rapid breathing. Abdominal breathing changes the breathing pattern, increasing oxygen intake and improving oxygen supply to the brain and body, which helps to relax the mind and body and reduce anxiety. During the training process, individuals focus their attention on their breathing, shifting their focus away from anxiety sources and reducing the interference of anxious thoughts, further alleviating anxiety.

### 4.2 Effects of abdominal breathing on physiological indicators

The experimental group showed lower heart rate, respiratory rate, and blood pressure after abdominal breathing training, indicating that abdominal breathing has a significant regulatory effect on physiological indicators during anxiety states. Heart rate and respiratory rate are important indicators reflecting the body’s stress state. During anxiety, sympathetic nerve excitement leads to increased heart rate and respiratory rate (Lehrer and Gevirtz, 2014). Abdominal breathing regulates the autonomic nervous system, reducing sympathetic nerve excitability and thus normalizing heart rate and respiratory rate (Shaffer and Ginsberg, 2017). Changes in blood pressure are also related to autonomic nervous system regulation and improvement in the body’s stress state. After abdominal breathing training, the body’s stress level decreases, vascular tension is reduced, and blood pressure is controlled to a certain extent (Russo et al., 2017). The improvement of these physiological indexes further explains the relieving effect of abdominal breathing on anxiety, and also suggests that abdominal breathing may have certain preventive and auxiliary therapeutic value for anxiety-related cardiovascular diseases.

### 4.3 Limitations and prospects

This study has certain limitations. The observation period was relatively short, only including 8 weeks of intervention effects and a 4-week follow-up after the intervention ended, with a lack of further research on the long-term effects of abdominal breathing training. The study subjects were limited to anxious individuals seeking help at a psychological counseling center, with relatively limited sample representativeness. Future research could expand the sample size to include different populations to enhance the generalizability of the results. The study did not delve deeply into the specific methods and frequency of abdominal breathing training, as different training methods and frequencies may yield varying effects on anxiety improvement. Subsequent research could further optimize the abdominal breathing training program. Despite these limitations, this study provides some basis for the application of abdominal breathing in improving anxiety. Future research could build on this study to conduct multi-center, large-sample, long-term randomized controlled trials to explore the mechanisms of abdominal breathing and the optimal intervention strategies, providing stronger support for the prevention and treatment of anxiety. Additionally, abdominal breathing could be combined with other treatments such as psychological therapy and medication to explore comprehensive treatment models, aiming to improve anxiety treatment outcomes.

## 5 Conclusion

This study indicates that abdominal breathing training can effectively improve anxiety states, reduce scores on the anxiety self-assessment scale, while regulating physiological indicators such as heart rate, respiratory rate, and blood pressure, and the intervention effects to some extent have persistence. As an intervention method that is simple, easy to implement, and free of side effects, abdominal breathing has certain clinical application and promotional value, and can be used as one of the auxiliary treatment methods for anxiety to help individuals with anxiety alleviate symptoms and improve quality of life.

## Data Availability

The original contributions presented in the study are included in the article/supplementary material, further inquiries can be directed to the corresponding authors.
